# Latitudinal variation in sexual dimorphism in life‐history traits of a freshwater fish

**DOI:** 10.1002/ece3.2658

**Published:** 2016-12-20

**Authors:** Satu Estlander, Kimmo K. Kahilainen, Jukka Horppila, Mikko Olin, Martti Rask, Jan Kubečka, Jiří Peterka, Milan Říha, Hannu Huuskonen, Leena Nurminen

**Affiliations:** ^1^ Department of Environmental Sciences/Aquatic SciencesUniversity of HelsinkiHelsinkiFinland; ^2^ Natural Resources Institute FinlandJyväskyläFinland; ^3^ Biological CentreAcademy of Sciences of the Czech RepublicHydrobiological InstituteČeské BudějoviceCzech Republic; ^4^ Department of BiologyUniversity of Eastern FinlandJoensuuFinland

**Keywords:** Bergmann's rule, growth, perch, Rensch's rule, sex, sexual maturity

## Abstract

Sexual dimorphism is common across the animal kingdom, but the contribution of environmental factors shaping differences between the sexes remains controversial. In ectotherms, life‐history traits are known to correlate with latitude, but sex‐specific responses are not well understood. We analyzed life‐history trait variation between the sexes of European perch (*Perca fluviatilis* L.), a common freshwater fish displaying larger female size, by employing a wide latitudinal gradient. We expected to find sex‐dependent latitudinal variation in life‐history variables: length at age, length increment, and size at maturity, with females showing consistently higher values than males at all latitudes. We further anticipated that this gender difference would progressively decrease with the increasingly harsh environmental conditions toward higher latitude. We hypothesized that growth and length increment would decrease and size/age at maturity would increase at higher latitudes. Our results confirmed female‐biased sexual size dimorphism at all latitudes and the magnitude of sexual dimorphism diminished with increase in latitude. Growth of both sexes decreased with increase in latitude, and the female latitudinal clines were steeper than those of males. Hence, we challenge two predominant ecological rules (Rensch's and Bergmann's rules) that describe common large‐scale patterns of body size variation. Our data demonstrate that these two rules are not universally applicable in ectotherms or female‐biased species. Our study highlights the importance of sex‐specific differences in life‐history traits along a latitudinal gradient, with evident implications for a wide range of studies from individual to ecosystems level.

## 1 Introduction

Size difference between the sexes is a common phenomenon among animals, but pronounced inter‐ and intraspecific variation exists in the magnitude of sexual size dimorphism (SSD) (Blanckenhorn, Stillwell, Young, Fox, & Ashton, 2006; Cox, Barrett, & John‐Alder, 2008). Several theories have attempted to explain the variation in SSD by different factors including sex‐dependent differences in sexual selection, vulnerability to predators, niche segregation, and parental investment (Abouheif & Fairbairn, 1997; Rennie et al., 2008). The regulation of SSD is complex because each of these factors may constrain or amplify the degree of dimorphism (Shine, 1989), and it is still unclear how these different factors determine the variation in sexual dimorphism (Mandiki et al., 2004; Young, 2005). One prominent macroecological pattern is the Rensch's rule (Rensch, 1950), which states that the magnitude of SSD tends to increase with increase in body size when males are the larger sex and to decrease with increase in size when females are larger (Fairbairn, 1997). Consequently, male body size varies more than female body size, irrespective of which sex is larger. Rensch's rule holds for a variety of animal taxa, for example, insects, reptiles, birds, and mammals (Blanckenhorn, Meier, & Teder, 2007; Fairbairn, 1997). However, the mechanisms underlying Rensch's rule remain obscure, and the rule appears to be more consistent in taxa with male‐biased SSD than in taxa with female‐biased SSD (Webb & Freckleton, 2007).

A general assumption is that response of sexes is similar to changes in environment, but some studies have shown differential sensitivity of males and females to environmental factors such as temperature (Fairbairn, 2005), thus potentially promoting variation in SSD. When environmental conditions improve, the sex that is more sensitive may achieve optimal size more readily than in poorer conditions resulting in an increase in SSD (Vedder, Dekker, Visser, & Dijkstra, 2005), hence, consistent or counter to Rensch's rule depending which sex is more sensitive. For example, large individuals require more food to attain larger size and to maintain body functions (Blanckenhorn, 1998), and are likely more sensitive to thermal variation via their higher metabolic rates (Pörtner & Peck, 2010). Thus, this follows that changes in the environment, such as latitudinal variation, may have a greater impact on the larger sex in SSD‐displaying species.

While Rensch's rule explains the relationship between body size and extent of SSD, another well‐known ecological rule, Bergmann's rule (Bergmann, 1848; ref. in James, 1970) describes geographical size variation. In addition, as Rensch's rule depends on body size variation, it has been suggested that Bergmann's (or converse Bergmann's) rule may relate to sexual size differences and their putative selective causes (Blanckenhorn et al., 2006). Bergmann's rule states that the body size of a widely distributed animal clade increases with latitude. While the direct applicability of Bergmann's rule is unestablished (Blanckenhorn et al., 2006; Meiri, 2011), the consensus is that latitudinal body size variation is evidently connected to temperature (Blanckenhorn & Demont, 2004). The usual explanation tendered for Bergmann's rule is that large animals expend less energy for thermoregulation, because of their small surface‐to‐volume ratio, and therefore, larger individual size is favored in colder climates. Bergman's rule was initially formulated for endothermic animals, and its extrapolation to ectotherms is controversial (Ashton & Feldman, 2003). In fact, opposite clines in body sizes (i.e., converse Bergmann's rule) are common in many ectotherms, such as frogs and salamanders (Adams & Church, 2008; Miaud et al., 2001), and in several fish species, body size decreases toward the poles (New, Hulme, & Jones, 1999; Vázquez & Stevens, 2004). In ectotherms, such as fish, the temperature‐associated shorter growing season at higher latitudes may limit body size (Blanckenhorn & Demont, 2004). However, Bergmann's rule for fish is still relatively under studied (Rypel, 2014).

In terms of growth or body size plasticity, fish are an interesting group because fish display allometric growth, which enables a faster response to changing environmental conditions relative to many endothermic animals (Arnold, Ruf, & Kuntz, 2006; Wootton, 2012). Temperature is the most important environmental variable governing metabolic activity (Brown, Gillooly, Allen, Savage, & West, 2004) and induces considerable phenotypic plasticity in body size of ectothermic animals (Angilletta & Dunham, 2003). Generally, growth of fish increases with increase in temperature to a species‐specific optimum value, decreasing thereafter (Wootton, 2012). Optimal temperature for growth may change with age and size, as juveniles generally prefer higher temperatures than adults (Pedersen & Jobling, 1989).

Teleost fish species display predominantly female‐biased SSD (Webb & Freckleton, 2007). As the gonad size of females generally increases more rapidly with size than that of males (Henderson, Trivedi, & Collins, 2000), large females are especially important in population‐level reproduction (Olin et al., 2012; Venturelli et al., 2010). According to the fecundity advantage hypothesis (Darwin, 1871; Shine, 1978), female‐biased SSD is due to selection favoring a large body size to ensure higher reproductive success, which also leads to the inverse of Rensch's rule (Fairbairn, 1997).

Here, we analyze the latitudinal variation in sexual dimorphism in life‐history traits in European perch (*Perca fluviatilis* L.) by using a comprehensive field data from core distribution (50ºN) to the northern distribution limit (69ºN). Perch is one of the most common freshwater fish species across Europe (38–69°N), inhabiting lentic habitats from ponds to the largest lakes (Kottelat & Freyhof, 2007). It is a cool‐water spring‐spawning species with an optimum growing temperature of ca. 23°C (Mélard, Kestemont, & Grignard, 1996) and a maximum length of 60 cm, but more typically attaining a length of up to 25 cm depending on lake and population type (Kottelat & Freyhof, 2007). The general life‐history traits of perch are well known and documented in several papers (Heibo, Magnhagen, & Vøllestad, 2005; Le Cren, 1951; Thorpe, 1977). Perch displays female‐biased sexual dimorphism in size, growth, and maturation (Heibo & Magnhagen, 2005; Mélard et al., 1996), but the sex‐specific latitudinal patterns are largely unknown.

We expected to confirm sexual dimorphism of growth and maturity of perch at all studied latitudes. As the energy demand of female perch is higher than that of males (Malison, Best, Kayes, & Amundson, 1985), in addition to the higher sensitivity of females to thermal variation (Estlander et al., 2015), we expected females to show a steeper latitudinal variation in growth. Thus, this follows that sex‐specific differences in growth and size would decrease toward higher latitude and would produce a pattern of SSD contrary to the prediction of Rensch's rule. Finally, possible explanations for observed patterns of SSD are discussed.

## 2 Materials and Methods

### 2.1 Sampling

Data were collected from 25 wild perch populations along a latitudinal gradient (50°–69°) with multimesh gillnets (European Standard Gillnet Sampling EN 14757; mesh range 5.25–60 mm) during 2000–2012 at the end of the growing season (August‐September) (Table 1). Both littoral and pelagial were sampled to assess the putative presence of divergent perch morphs (Svanbäck & Eklöv, 2003). However, we did not find signs of perch population divergence. The data included 2736 individuals: 1139 males (42%) and 1597 females (58%) (Table 1). At latitudes from 69 to 50°N, the annual average water temperatures increase from 2 to 9°C (Straškraba, 1980) and the length of the growing season from 110 to 190 days (Rötzer & Chmielewski, 2001). Latitude, longitude, altitude, lake surface area, and total phosphorus concentration were measured from all study lakes. Water quality parameters, such as water transparency, that is, Secchi depth (average of all lakes, 3.6 m ± 2.2 *SD*) and pH (6.9 ± 0.3 m) varied, but were not statistically significant between latitudes (*p* < .05). All the sampled lakes are multispecies, and species diversity and fish density vary both between lakes and latitudes.

**Table 1:** Summary of latitudinal perch mean size and age (±*SD*) data used in analyses (CZE= Czech Republic, SFI= Southern Finland, CFI=Central Finland, NFI=Northern Finland). Lake characteristic values represent the mean values per latitude, with a range in parentheses

Population origin				Lake information				Fish data metrics			
Latitude (ºN)	Longitude (ºE)	Altitude (m a.s.l.)	Populations (*n*)	Lake size (ha)	Mean depth (m)	TotP (μg L^−1^)	Perch Individuals (*n*)	Female (%)	Total length (mm)	Weight(g)	Age (years)
50	16	240	4 (CZE)	120 (60‐250)	17 (14‐23)	44 (10‐80)	668	58	180 ± 2	96 ± 5	4 ± 0.6
60	25	130	6 (SFI)	270 (20‐700)	5 (3‐6)	21 (5‐40)	516	60	150 ± 2	51 ± 3	3 ± 0.9
63	29	150	6 (CFI)	230 (20‐470)	4 (4‐5)	10 (5‐14)	350	62	160 ± 3	63 ± 4	4 ± 0.1
69	26	200	9 (NFI)	13660 (350‐104300)	8 (3‐14)	7 (4‐21)	1202	56	193 ± 2	109 ± 3	6 ± 0.5

### 2.2 Length increment, maturity, and sexual size dimorphism

Sex, total length (accuracy 1 mm), and weight (0.1 g) were measured, and opercula were cleaned for age and back‐calculated growth determinations (Bagenal & Tesch, 1978). The length or age at maturity data was not available for all of the lakes studied, but length and age at maturity are known to correlate positively with latitude in perch (Heibo, 2003; Heibo et al., 2005). As the data analyzed here showed a similar pattern, age at maturity was estimated according to Heibo (2003) by linear regression: age at maturity = −1.2 + 0.04 × latitude; *R* ^*2*^ = .59, *p *<* *.005, and these were used to assess the length at maturity from current data. The back‐calculated growth of perch was determined from the otolith or operculum bone for each individual using the Monastyrsky method: nonlinear relationship between the otolith/operculum radius and total length of the fish (Bagenal & Tesch, 1978): *L*_*i*_ *= (S*_*i*_ */S*_*c*_ *)* ^*b*^ *× L*_*c*_,

where *L*_*i,*_ *S*_*i*_ = length of fish at formation of i:th radius or radius at age *i*;* L*_*c*_ *, S*_*c*_ = length of fish or radius at the time of capture; and *b *= growth coefficient i.e. the slope of the relationship between otolith/operculum radius and length.

The between‐sex and latitudinal differences in the annual (a) length increments and (b) length at age were analyzed with analysis of variance for repeated measures (ANOVAR), and data used were restricted to age groups 1–6 (*n* = 2004), because older fish were rare or absent from southern populations (50°). In the ANOVAR models, sex (two levels) and latitude (four levels) were considered as fixed factors. Mauchly's test indicated that the assumption of sphericity had been violated in both models, (a) $x_{\left(14\right)}^{2}=5699.44$, *p *>* *.05; (b) $x_{\left(14\right)}^{2}=332.44$, *p *>* *.05, and therefore, degrees of freedom were corrected (e.g., Field, 2013) using Greenhouse–Geisser estimates of sphericity (a) (*p *=* *.280); (b) (*p *=* *.872).

Ratios for sexual dimorphism indices (SDI) were calculated using the method of Gibbons (1992):

(A/B)−1,

where A is the mean size of the largest sex and B is the mean size of the smallest sex. Sex‐specific mean sizes for SDI calculation were weighted by number of individuals within age groups (2–10 years) when lake‐specific subsamples represented true length and age distribution in each lake; thus, one SDI value per population (in total 25) was calculated. Stepwise multiple regressions with forward selection of variables were used to identify the most important environmental variables explaining the variation in SDIs. Environmental variables (latitude, longitude, altitude, lake surface area, and total phosphorus concentration) were entered in the multiple regression analysis, if *p *<* *.05. The statistical analyses were performed using IBM SPSS Statistics for Windows, version 21.0 (IBM Corp., Armonk, NY, USA).

## 3 Results

### 3.1 Growth

**Figure 1:**
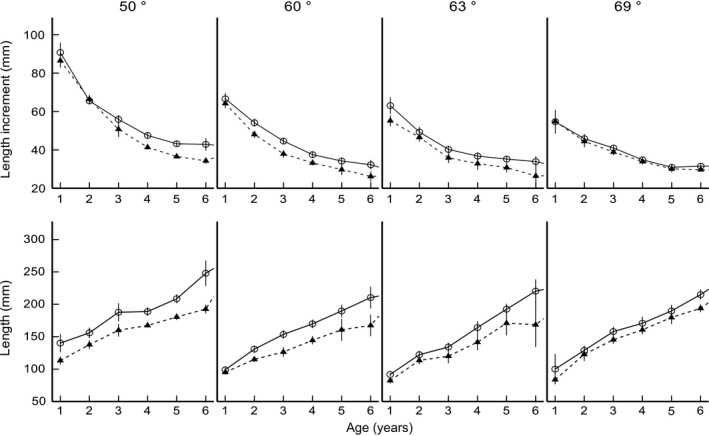
Average annual total length increments (top) and average total length at age (bottom) with standard deviation (±*SD*) of female (open circles) and male (black triangles) perch at latitudes 50–69°N

**Figure 2:**
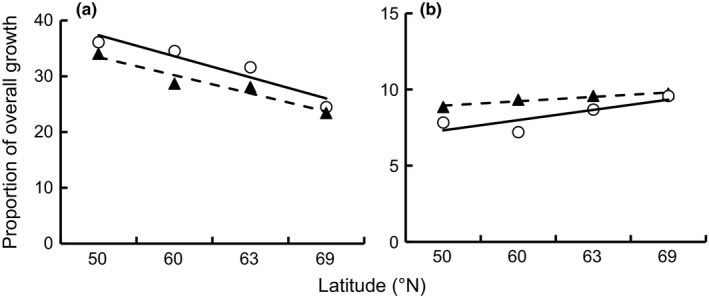
Proportion of overall growth (total length interment) of female (open circles) and male (black triangles) perch in the first year (a) and fifth year (b)

The latitudinal variations of length at age (ANOVAR; *F*[_3,924_] = 6.92, *p *<* *.001) and length increment (ANOVAR; *F*[_3,924_] = 2.10, *p *=* *.010) of perch were sex‐dependent (Figure 1). Overall, the growth of perch decreased with increase in latitude (ANOVAR; *F*[_3,924_]* *= 131.07, *p *<* *.001), and the length at age of females was larger than that of males at all latitudes (ANOVAR; *F*[_1,924_] = 36.48, *p *<* *.001) (Figure 1). However, the length at age and length increment difference between sexes varied, depending on latitude and age (Figure 1; Table 2). At latitude 50°N, the length at age for females was larger than for males in all age groups (ANOVAR; *F*[_5,384_] = 14.99, *p *<* *.001) (Table 2), and the annual length increment was higher in females than in males (ANOVAR; *F*[_1,84_]* *= 14.38, *p *<* *.001), despite no sex‐dependent differences in age groups 2 and 6 (Table 2; Figure 1). At latitude 60°N, females were overall larger than males (ANOVAR; *F*[_5,76_]* *= 5.54, *p *=* *.012), indicated by sex‐dependent differences in age groups 2–6 (Table 2; Figure 1). No significant sex dependency on annual length increments in the latitude 60ºN increment was detected when pooling all age groups (ANOVAR; *F*[_1,76_] = 3.02, *p *=* *.086); however, a sex‐dependent difference was observed in age groups 4 and 5 (Table 2), with growth of females being faster than that of males (Figure 1). At latitude 63°N, females were larger (ANOVAR; *F*[_5,106_] = 4.72, *p *=* *.02) and grew faster (ANOVAR; *F*[_1,106_] = 4.57, *p *=* *.035) than males when all age groups were pooled, indicated by sex‐dependent differences in length at age and length increment in the older age groups (groups 4–6) (Table 2; Figure 1). When all age groups were pooled at the northernmost latitude 69°N, females were larger (ANOVAR; *F*[_5,658_] = 5.26, *p *=* *.012) and grew faster (ANOVAR; *F*[_1,658_] = 4.09, *p *=* *.043) than males (Figure 1), indicated by sex‐dependent differences in length at age in age groups 5 and 6 (Table 2) and the annual length increment in age groups 1, 3, 5, and 6 (Table 2). In general, the annual length increment showed disparate latitudinal clines; the southernmost populations had the fastest growth in the early years of life and the northernmost populations in later years (Figure 2a,b). Additionally, in the first year of life, a greater proportion of overall growth was attained by females relative to males, whereas in older individuals, the opposite trend was observed (Figure 2a,b).

**Table 2:** *p*‐values from repeated measurements analysis of variance in between‐sex comparisons of annual total length increments and length at age of perch at latitudes of 50–69°N. Significant values (*p* < .05) are in bold

Age (years) and sample size	Latitude (°N)							
	50°	60°	63°	69°	50°	60°	63°	69°
	Annual length increments				Length at specific age			
1_(*n* = 292)_	**0.0323**	0.6873	0.1878	**0.0158**	**0.0323**	0.6873	0.1878	0.2050
2_(*n* = 293)_	0.2492	0.4996	0.6628	0.2121	**0.0224**	**<0.0001**	0.2193	0.6220
3_(*n* = 316)_	**<0.0001**	0.1538	0.2625	**0.0473**	**0.0007**	**<0.0001**	0.1484	0.2850
4_(*n* = 485)_	**0.0009**	**0.0274**	**0.0220**	0.1588	**<0.0001**	**<0.0001**	**0.0436**	0.1190
5_(*n* = 350)_	**0.0026**	**0.0213**	**0.0232**	**0.0190**	**<0.0001**	**0.0010**	**0.0383**	**0.0406**
6_(*n* = 260)_	0.0735	0.1568	**0.0063**	**0.0058**	**<0.0001**	**0.0290**	**0.0132**	**0.0105**

When pooling all age groups and both sexes, the maximum length of perch decreased with increase in latitude (420, 365, 344, and 332 mm at latitudes 50, 60, 63, and 69°N, respectively). The average length varied significantly between latitudes (ANOVA, *F*[_3,2736_]* *= 95.59, *p *<* *.001), but without a clear latitudinal trend (Table 1).

### 3.2 Longevity and maturity

The average age varied significantly between all latitudes (ANOVA, *F* [_3,2736_] = 177.53; *p *=* *.001), as the oldest fish were found at latitude 69°N and youngest at latitude 60°N (Table 1). Males were 1.2–1.5 years younger than females at latitudes 60, 63, and 69°N (ANOVA; *F*[_3,2728_] = 17.69, *p *<* *.001), but at latitude 50°N, the average age of females and males was the same. The age at maturity increased from 2 to 5 years with increase in latitude, and the corresponding length at maturity increased ca. 50 mm for both males (126–180 mm) and females (138–190 mm) (Table 3).

**Table 3:** Age at maturity (±*SD*) at different latitudes estimated according to Heibo (2003) by linear regression and the corresponding total lengths for these ages analyzed from data

Latitude (°N)	Age at maturity (years)	Length at maturity (mm)	
		Males	Females
50°	2 ± 0.18	131 ± 17	138 ± 21
60°	3 ± 0.21	126 ± 21	154 ± 24
63°	4 ± 0.27	142 ± 20	164 ± 25
69°	5 ± 0.18	180 ± 38	190 ± 41

### 3.3 Sexual size dimorphism

**Figure 3:**
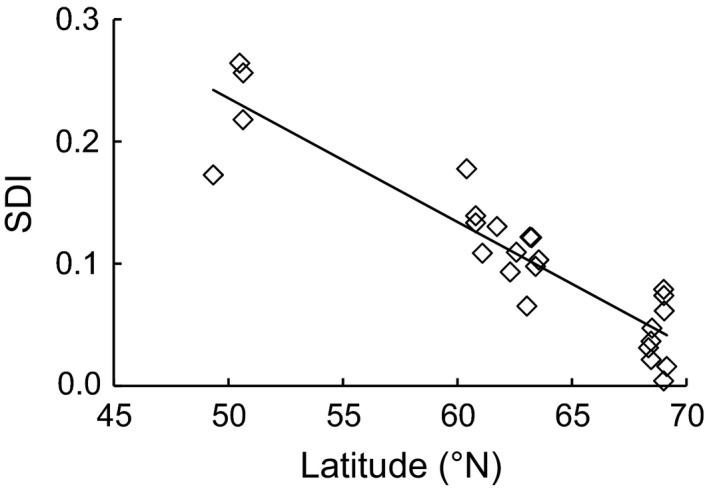
Latitude‐specific degree of sexual size dimorphism in perch populations (SDI = −0.01 × latitude + 0.731, *R* ^2^ = .84, *p* < .0001)

In the stepwise multiple regression model, other environmental factors failed to enter the regression equation once latitude was included (Table 4). The degree of sexual size dimorphism of perch decreased with increase in latitude, and latitude significantly predicted *SD* indices (Figure 3) (*R* ^2 ^= .84; *F *=* *93.33; *p *<* *.001), indicating that the size difference between sexes was inversely associated with latitude.

**Table 4:** Stepwise multiple regression model (factors included when *p* < .05) for sexual size dimorphism. The only factor selected was latitude

	β	*t*	*p*
Latitude	0.731	13.561	.0001
Longitude	−0.018	−0.142	.889
Altitude	−0.146	−1.614	.122
Lake size	−0.149	−1.466	.158
Total phosphorus	−0.214	−1.762	.093

## 4 Discussion

As expected, all perch populations studied exhibited significant sexual dimorphism in growth, size, and maturity, with females growing larger and maturing later than males. Growth of both sexes decreased and the length at maturity increased with latitude, but latitudinal trends were generally steeper in females than in males. Accordingly, the magnitude of SSD diminished in concert with increase in latitude, suggesting stronger sensitivity of females to latitudinal variation, because female body size showed an increased plasticity relative to males. Thus, perch did not follow Rensch's rule in the present study, but showed the exact converse pattern. In contrast, our results are consistent with the conception that growth response can be sex‐specific to environmental conditions (Bonduriansky, 2007; Stillwell, Blanckenhorn, Teder, Davidowitz, & Fox, 2010). In addition, studies that describe the inverse of Rensch's rule (e.g., Fairbairn, 1997) suggest that SSD results from fecundity selection favor larger female size. This likely holds also for perch, because the fecundity of perch increases with female body size (Olin et al., 2012).

Several factors inducing gender‐specific differences in growth have been proposed, including energy allocation, risk‐taking, and vulnerability to predators and parental investment (Rennie et al., 2008). More recently, sex‐specific differences of perch have been observed in gut microbiota linked to distinct dietary preferences (Bolnick et al., 2014) and may thus have further implications of energy routing and individual metabolism. Due to the different demands for energy acquisition, males and females may have variable strategies for trade‐offs between food acquisition and prevailing environmental conditions (Holtby & Healey, 1990). In optimal environmental conditions (e.g., clear water for foraging, optimal temperature, low predation pressure), females invest in active feeding to ensure somatic growth and later gonadosomatic growth, whereas males do not need to invest as much in feeding and fast growth, as sperm is less energy‐demanding to produce (Rennie et al., 2008). Therefore, changes in optimal feeding conditions have the most pronounced effects on the most active feeders, which are often females (Estlander et al., 2015; Horppila et al., 2011), whereas males need to grow only to size at sexual maturity. Consequently, contradicting with Rensch's rule which implies that sexual selection is the main driving force underlying SSD (Fairbairn, 2005), we suggest that different forces beyond sexual selection, such as sex‐specific responses to variation in environmental conditions, may be also responsible for shaping SSD patterns in perch.

In addition to latitudinal variation in SSD, perch displayed an overall decreasing growth in terms of length increment with increase in latitude irrespective of sex. This is in line with Heibo et al. (2005), who suggested that perch follow the converse pattern of Bergmann's rule in growth. Many animal taxa, such as birds in general, have a strong intraspecific tendency toward larger body sizes at higher latitudes and in cooler environments (Ashton, 2002), in contrast to many ectotherms (Angilletta, Steury, & Sears, 2004). Corroborating our results, Heibo et al. (2005) found many life‐history variables, such as length at age and length increment to decrease and age at maturity to increase with latitude. This is attributed to the latitudinal cline in temperature and duration of the growing season. Also supporting our results, the asymptotic body length did not similarly correlate with latitude. In our data, the growth of perch decreased while the average length and age increased along latitude, suggesting a greater longevity of northern populations. Large size can be the result of greater longevity, if mortality is low even with relatively slow growth (Angilletta et al., 2004). In cooler climates at higher latitudes, fish may invest more in somatic growth to reach a larger size at the expense of gonad growth, suggesting a trade‐off between individual energy allocations. Year‐class strength of perch populations at distribution limit is also known to be highly dependent on temperature (Hayden, Harrod, & Kahilainen, 2014; Tolonen, Lappalainen, & Pulliainen, 2003) that may also promote subsequent growth of single year class to large size in multispecies communities. In general, perch populations consist of larger sized individuals in higher latitudes (Jeppesen et al., 2010), suggesting that the population‐level shift to piscivory maybe more frequent than in lower latitudes or merely reflects lower temperature‐related metabolic costs and thus higher longevity in north. Also, other biotic factors, not considered here, such as available food resources, intraspecific competition and interspecific competition affect growth of perch. However, latitude (temperature, duration of the growing season, productivity) directly and indirectly regulates several abiotic and biotic factors and therefore potentially also affect the trophic interactions between species (Jeppesen et al., 2010). Accordingly, our results suggest that in addition to sex‐specific sensitivity to environment, variation in SSD could result from sex‐specific differences in longevity, age structure, or differences in diet. Further field and experimental studies combining sex‐specific dietary, size structure, and life‐history trait data are needed to assess these patterns.

Some studies have suggested that different timing of maturity between the sexes may be responsible for the level of SSD exhibited by a species (Fairbairn, 1990; Gibbons & Lovich, 1990). These studies imply that juvenile growth rates between the sexes are similar, and the earlier maturing sex remains smaller than the later maturing sex (Badyaev, 2002). In this study, males matured earlier than females, a pattern common in fish, as females increase their fecundity with size, but reproductive success in males is not as size‐dependent (Stearns, 1992). However, Blanckenhorn et al. (2007) suggested in a study with insects that SSD is more likely related to differential growth rates between the sexes that may be differently constrained by growth conditions when attaining their optimal body sizes and the larger sex shows stronger response to a reduction in environmental quality. The results of our research support this suggestion, because perch showed a sex‐dependent difference in size and back‐calculated growth already at the juvenile stage, as females appeared to invest more in growth in the early years of life. Faster growing fish are also more likely to shift to piscivory that maybe more important for females benefiting on larger maturity size more than males. This could be an important mechanism to understand bimodal size structure of perch populations as well as prey fish communities, but remains to be evaluated in experimental and field studies. Therefore, we argue that growth rate, rather than timing of maturity, maybe a more significant factor behind SSD variation in perch. It must be noted, however, that it is difficult to rank these two factors in order of importance, as these life‐history traits are highly correlated (Stearns, 1992).

There is a linear relationship with latitude and temperature, and rising temperature accelerates growth and earlier maturity (Berrigan & Charnov, 1994; Heibo et al., 2005), demonstrated also in this study, as the maturation length and age at maturity increased with latitude in both sexes. Typically, delayed maturation provides a benefit, because fecundity increases with body size (Roff, 2002; Stearns, 1992). According to Heibo et al. (2005), the maximum reproductive life span, that is, higher longevity increases with latitude in perch, but reproductive investment (measured as relative gonad mass) in each spawning season decreases with latitude. Such life‐history strategy is beneficial, if mortality is low, that is, reproductive life span of both sexes is long. Similar findings are suggested also in coho salmon (*Oncorhynchus kisutch*) as the egg number increases, but the individual egg size and the total egg biomass decrease with latitude, and thus, at the population level, the gametic effort may be constant with latitude (Tamate & Maekawa, 2006).

Overall, the regulation of SSD is a complicated issue and widely accepted ecological rules such as Rensch's and Bergmann's rules describing the patterns in body size are not straightforwardly applicable in fish species with female‐biased SSD. For instance, even if fecundity selection would be the ultimate cause behind the SSD of fish, several environmental factors might regulate the magnitude of SSD, such as latitude that potentially regulates a complex mix of environmental and ecological factors. Moreover, sensitivity to these factors may vary between sexes. In addition, it is difficult to distinguish whether phenotypic changes in growth or size/age structure along a latitude gradient are a result of adaptive evolution or phenotypic plasticity or a mixture of both (e.g., Kuparinen & Merilä, 2007), and thus, more research is needed, such as common garden experiments, to better understand both the phenotypic and genetic relationships between SSD and growth. Our research demonstrates latitudinal population‐level variation in the magnitude of SSD based on growth rate, supporting the predictions of previous experimental and regional studies by Fontaine, Gardeur, Kestemont, and Georges (1997), Horppila et al. (2011), and Estlander et al. (2015), all suggesting that environmental factors limiting overall growth may decrease the magnitude of SSD. This study also highlights the importance of sex‐specific response differences to environmental variables in regulating patterns of allometry between the sexes in fish. In general, understanding the causes behind body size variation is particularly important in fish as it is related to fecundity and survival. Our results of growth and sexual maturity of an abundant fish in European lakes suggest that sex has an important role in determining life‐history traits, but may have implications on individual metabolism, predator–prey relationships, and size structuring of fish populations in lakes. We conclude that follow‐up studies from individual to ecosystem level scale are needed to assess potentially holistic consequences of sexual size dimorphism.

## Conflict of Interest

None declared.
